# Sparking potential over 1200 V by a falling water droplet

**DOI:** 10.1126/sciadv.adi2993

**Published:** 2023-11-15

**Authors:** Luxian Li, Xuemei Li, Wei Deng, Chun Shen, Xinhai Chen, Han Sheng, Xiang Wang, Jianxin Zhou, Jidong Li, Yinlong Zhu, Zhuhua Zhang, Jun Yin, Wanlin Guo

**Affiliations:** ^1^ State Key Laboratory of Mechanics and Control of Mechanical Structures, Key Laboratory for Intelligent Nano Materials and Devices of the Ministry of Education, Nanjing University of Aeronautics and Astronautics, Nanjing 210016, P. R. China.; ^2^ College of Aerospace engineering, Nanjing University of Aeronautics and Astronautics, Nanjing 210016, P. R. China.; ^3^ College of Material Science and Engineering, Nanjing University of Aeronautics and Astronautics, Nanjing 210016, P. R. China.; ^4^ Institute for Frontier Science, Nanjing University of Aeronautics and Astronautics, Nanjing 210016, P. R. China.

## Abstract

Hydrovoltaic technology has achieved notable breakthroughs in electric output via using the moving boundary of electric double layer, but the output voltage induced by droplets is saturated around 350 volts, and the underlying mechanism remains to be further clarified. Here, we show that falling water droplets can stably spark an unprecedented voltage up to 1200 volts within microseconds that they contact an electrode placed on top of an electret surface, approaching the theoretical upper limit. This sparking potential can be explained and described by a comprehensive model considering the water-electrode contact dynamics from both the macroscale droplet spreading and the microscale electric double layer formation, as well as the presence of a circuit capacitance. It is demonstrated that a droplet-induced electric spark is sufficient to directly ionize gas at atmospheric pressure and split water into hydrogen and oxygen, showing wide application potential in fields of green energy and intelligence.

## Teaser

A single falling droplet can stimulate a voltage output up to kilovolts, paving the way for high-voltage applications.

## INTRODUCTION

In a hybrid liquid-solid system containing charge carriers of ions, local electric potentials can be changed by ion migration through confined channels, such as action potential of a pulsed spike in our nervous system (*1*, *2*) and the electrokinetic effect of streaming potential (*3*). However, because of the speed at which the ions move, the rise in potential often takes a few milliseconds, leading to a restrained voltage amplitude up to hundreds of millivolts.

Recently, hydrovoltaics (*4*–*6*) becomes a burgeoning field in harvesting water energy. It relies on direct interactions between functional materials and variable forms of water, namely, falling (*7*), flowing (*8*, *9*), waving (*10*), and even evaporating (*11*) water. Among them, hydrovoltaic energy generation from falling water droplets has attracted much interest because of the wide distribution of daily rainfall and tremendous energy embedded in (*12*), which has long been wasted. Electrical energy harvesting from water droplets can be date back to Lord Kelvin’s water-drop electrostatic generator (*13*), which uses falling water droplets to generate a voltage by electrostatic induction occurring between interconnected, oppositely charged systems. However, notable output voltage requires the long-term accumulation of numerous charged droplets, preventing it from practical application. In contrast, hydrovoltaic technology can easily induce electricity through water droplet moving on solid surfaces. In 2014, Yin *et al.* (*14*) found that moving water droplets along or dropping them on a two-coplanar-electrode device consisting of a single-layer graphene supported on insulating substrate can generate electricity at the level of tens of millivolts. As the output voltage is proportional to the moving velocity of electric double layer (EDL) boundaries, the induced potential is termed as drawing potential, and a modified electrokinetic model with consideration of moving boundaries of EDL was developed (*15*). Waving water surface across graphene can also induce a potential gradient, verifying the developed model (*10*). Since then, intensive efforts have been dedicated to boosting the electric output induced by moving water and demonstrating creative applications. Through adapting polarized substrates or applying a biased voltage, the falling water droplet–induced drawing potential in the two-coplanar-electrode devices can be increased up to ~5 V due to the enhanced charge interaction at the solid-liquid interface (*7*, *16*, *17*).

Xu *et al.* (*18*) introduced a transistor-inspired device structure (*19*–*23*), which was referred to as droplet energy generator (DEG), that notably boosted the falling water droplet–induced voltage to around 140 V. Subsequently, many insightful methods, including surface charge injection (*24*), coplanar-electrode design (*25*), surface wetting modulation (*26*, *27*), etc., have been further developed (*28*–*32*). However, the output voltage appears to be saturated at around 350 V (*32*). Most of the works devoted into the optimization of DEG are based on an oversimplified model (*18*, *24*, *32*). Although it is commonly recognized that the voltage output was triggered by the contact between the water droplet and top electrode, it is unexpected to find that the dynamics of such a contact was fully ignored in the model. Evaluations on other factors inevitable in practice, such as the presence of parasitic capacitance, and their influence on the output performance are also lack. Comprehensive investigations on these factors could not only deepen our fundamental understanding on its working mechanism but also, from the practical aspects, pave its way to various high-voltage applications, such as instantaneous luminescence (*33*), electrospinning (*34*), tunable optical grating (*35*), and so on.

Here, we show that an unprecedented sparking potential of 1200 V can be achieved at the instant, while a spreading droplet touches the electrode. The dynamics in water/top-electrode contact is found to be critical in determining the output voltage. It is unveiled that, at the contact instant, a high sliding speed and a large spreading area of the impacting droplet at the macroscale, as well as a short relaxation time and a thin thickness of EDL at the microscale, are necessary to achieve the ultrahigh sparking potential. Moreover, we found that the presence of a circuit capacitance also played an important role. Benefiting from the comprehensive considerations of these two important factors and corresponding device optimization, kilovolt output voltage was firstly achieved in this work. Such an ultrahigh sparking potential can stimulate the ionization of helium gas directly at atmospheric pressure, producing notable microsparks and squeaks. Even for the case in connection with an electrolytic cell of notably large capacitive load, the droplet-induced sparking potential is high enough to split water, which is inaccessible for traditional devices. The achievement of droplet-induced sparking potential over 1 kV marks the entry of hydrovoltaic technology into the kilovolt era and opens a fresh vision for hydrovoltaics.

## RESULTS

**Fig. 1.**
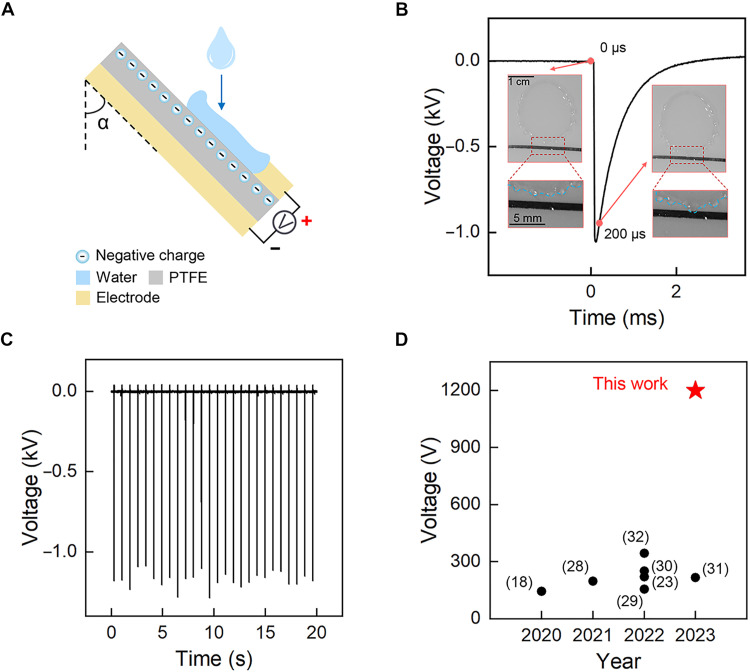
Device configuration and optimized performances. (**A**) The device configuration, not to scale, α denotes the inclined angle of the device. (**B**) A typical voltage peak signal of the sparking potential and corresponding snapshots just before and after the spreading droplet touches the top electrode. Interval time between two sequential snapshots is 200 μs. The blue dashed line indicates the droplet boundary. (**C**) Typical signals of optimized electric output. (**D**) Statistics of the output peak voltage for reported DEGs driven by falling water droplets.

In our experiments, devices with a configuration as illustrated in Fig. 1A are adopted (*18*, *26*). A photograph is shown in fig. S1. To evaluate the device performance, 60 μl of droplets of tap water, unless otherwise stated, were released from a 25-cm height above an inclined polytetrafluoroethylene (PTFE) surface, with a metallic strip acting as the top electrode and an Al tape as the bottom electrode. Voltage signals between these two electrodes were recorded. The PTFE surface is negatively charged with a density, *q*_0_, around −35 ± 3 μC/m^2^, which is determined through surface potential measurements (see Materials and Methods for details). A PTFE film with a thickness of 600 μm is chosen as the output voltage increases with film thickness (fig. S2).

High-speed camera observation in synchronization with the electric measurements reveals that a voltage peak signal is triggered once the spreading droplet crosses the electrode edge, as illustrated in the insets of Fig. 1B. The rising time of the voltage peak is only around 40 μs (fig. S3), in sharp contrast with the millisecond rising time of potential induced by ion migration. For a fixed amount of electricity to be released/converted, the shorter in duration, the higher amplitude in output voltage or current. In combination with optimization of the microsecond contact dynamics and the system circuit, as will be discussed below, an ultrahigh output voltage of 1200 V can be achieved steadily in our device, as shown in Fig. 1C. In view of the instantaneous and ultrahigh voltage, we term the voltage induced here by the falling droplet as sparking potential. So far, the performance of devices for harvesting energy from a single droplet has been successfully improved from volt level in drawing-potential devices to hundred-volt level in most DEGs and to kilovolt level in this work (see Fig. 1D).

**Fig. 2.**
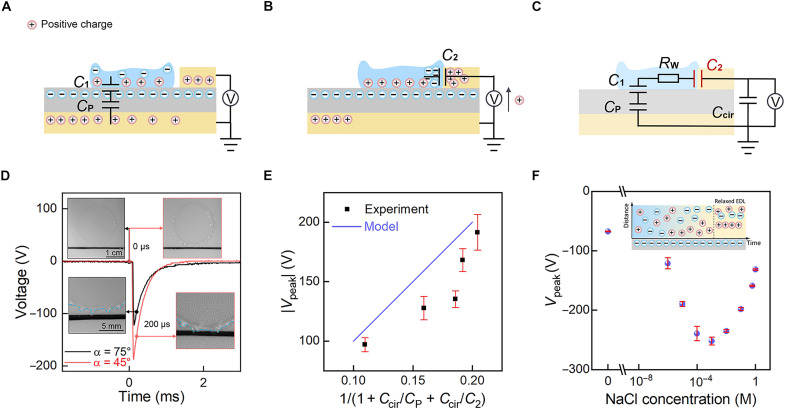
Working principles and optimization of the microsecond contact dynamics. (**A** and **B**) Schematic of charge distribution in the device before (A) and after (B) the spreading droplet touches the top electrode. (**C**) Equivalent electrical circuit of the device. (**D**) Typical output voltage signals for a device placed at α = 75° and 45° and corresponding snapshots just before and after the spreading droplet touches the top electrode. Interval time between two sequential snapshots is 200 μs. The blue dashed line indicates the droplet boundary. (**E**) |*V*_peak_| versus 1/(1 + *C*_cir_/*C*_P_ + *C*_cir_/*C*_2_), verifying the dependence of *V*_peak_ on the droplet dynamics. The determination of *C*_cir_, *C*_P_, and *C*_2_ can be found in Supplementary Text. (**F**) *V*_peak_ versus NaCl concentration, manifesting the dependence of *V*_peak_ on the dynamic characters of EDL. Note that the voltages measured here are for devices without *C*_cir_ optimization, thus relatively low.

In general, the working principle of the device can be described as following. While the falling droplet contacts the PTFE film, an EDL forms at the water/PTFE interface, which can be treated as a capacitor, *C*_1_. Meanwhile, the PTFE dielectric film and the bottom electrode compose another capacitor, *C*_P_, in which the water/PTFE serves as the top plate and PTFE/Al serves as the bottom plate. The falling and spreading processes of the droplet convert the mechanical energy to electrical energy at the solid-liquid interface, which is equivalent to charging *C*_1_ (Fig. 2A). Following, while the moving boundaries of the droplet touch the top electrode (Fig. 2B), another EDL forms at the water/top-electrode interface, giving rise to a capacitor, *C*_2_. The introduction of *C*_2_ would cause the release of stored electrical energy in *C*_1_ and *C*_P_ into *C*_2_ instantaneously and spark an electrical output as shown in Fig. 2D. After the spreading droplet reaches its maximum area, the shrinkage in droplet area, thus reduction of *C*_1_, drives a back flow of the charges between the top and bottom electrodes and gives rise to a positive peak voltage around 40 V (fig. S4). To analyze the whole process, an equivalent circuit is proposed as shown in Fig. 2C. The spreading water droplet was treated as a resistor, *R*_W_. In addition, in practical electrical measurements, there also exists a circuit capacitor, *C*_cir_. By analyzing the equivalent electrical circuit of the device (see the Supplementary Materials), we obtained that the output negative peak voltage

$$V_{\mathrm{peak}}=\frac{1}{1+C_{\mathrm{cir}}/C_{P}+C_{\mathrm{cir}}/C_{2}}\cdot U_{0}$$ (1)

where *U*_0_ is the initial voltage across *C*_P_ and *C*_2_ indicates the capacitance of water-electrode EDL capacitor at the moment that the output voltage arrives peak value. *C*_1_ has been ignored here, since the thickness of the PTFE film (600 μm) is several orders of magnitude larger than the Debye length of EDL (roughly 1 μm for deionized water and decreases with the increases in ion concentration) (*36*). Here

$$C_{P}=\frac{\varepsilon_{P}}{d}\cdot S$$ (2)

where *S* is the area of the spreading droplet while its moving boundaries just touch the top electrode and ɛ_P_ and *d* are dielectric constant of PTFE (2 ɛ_0_) and thickness of the PTFE (600 μm), respectively.

In the oversimplified circuit adapted widely (*18*, *24*–*26*, *32*), *C*_2_, as highlighted by red color in Fig. 2C, was also ignored. Under this hypothesis, the electrical output was believed to be dominated by the spreading area of the droplet as the amount of discharging charge is proportional to *C*_P_ (*26*, *27*). However, we note that the microsecond contact dynamics during the rise of voltage peak make the aforementioned hypothesis invalid, and time-varying character of *C*_2_ is important in determining the output *V*_peak_. The microsecond contact dynamics, manifesting in the equivalent circuit as formation of *C*_2_, involve two aspects, the dynamics of spreading droplets at the macroscale and relaxation of EDL at the microscale. In this way, *C*_2_ in Eq. 1 could be expressed as

$$C_{2}=t_{\mathrm{rising}}\frac{dS_{\mathrm{overlap}}}{dt}\cdot \frac{\varepsilon_{2}}{\lambda_{D}}\cdot \left(1-e^{\frac{-t}{\tau_{D}}}\right)$$ (3)

where *S*_overlap_ is the overlap area between the droplet and the top electrode, *t* and *t*_rising_ are contact time and the rising time of the instantaneous voltage signal, and λ_D_, τ_D_, and ɛ_2_ are Debye length (a character to describe the thickness of EDL, 10 nm) of *C*_2_, Debye time (a character to describe the relaxation time of EDL) of *C*_2_, and the dielectric constant of water (78 ɛ_0_), respectively. In addition to that, $\left(1-e^{\frac{-t}{\tau_{D}}}\right)$ is the typical relaxation form of *C*_2_ (*37*, *38*).

Regarding to the macroscale dynamics, since the capacitance of *C*_2_ is proportional to $t_{\mathrm{rising}}\frac{dS_{\mathrm{overlap}}}{dt}$, the overlap area between the droplet and the top electrode at the moment that the output voltage arrives peak value, the formation rate of *C*_2_ is determined by the moving speed of the droplet boundary. To tune the boundary moving speed, we simply adjust the inclined angle of the device, α. As shown in Fig. 2D, while reducing α from 75° to 45°, although the spreading areas *S* of droplets before touching the top electrode remain nearly the same, 243 and 255 mm^2^ for 75° and 45°, respectively, *V*_peak_ increases notably from ~120 to ~190 V. The enhancement of *V*_peak_ was attributed to the larger moving speed of the droplet boundary, as manifested by the larger overlap area of the droplet with the top electrode just 120 μs after they became in contact, as recorded by the high-speed camera (insets of Fig. 2D). The experimental results at different inclined angles (fig. S5) show the same trend as that predicted by Eq. 1, as demonstrated in Fig. 2E (*t*_rising_ = 40 μs and *C*_cir_ = 29.18 pF for the calculation; see figs. S3 and S8C). The deviation between the experimental data and theoretical results may be contributed to the dependence of PTFE surface charge density on the droplet film impacting intensity (*39*) and thus possibly the inclined angles of the device. Besides, the complex interplay between the resistor-capacitance (RC) circuit discharging dynamics of the whole device and the dynamic contact between droplet and top electrode was not considered in our model. In simple words, before the output voltage rises to the peak value, partial charges have been released through the circuit, leading to a reduced peak voltage compared to the predicted one.

Regarding to microscale, the formation rate of *C*_2_ also depends on the relaxation time and thickness of EDL, see Eq. 3, as manifested by ion concentration dependence of *V*_peak_. With the increase of NaCl concentrations from 0 to 1 mM, *V*_peak_ increases notably from 70 to 250 V, as shown in Fig. 2F. It is contributed to the reduced Debye length and Debye time with the increase in ion concentrations (*37*, *40*). With the NaCl concentration increasing from 0 to 1 mM, the Debye length [$\lambda_{D}={\left(\frac{\varepsilon k_{B}T}{\sum_{i}{\left(c_{i}\right)}_{0}z_{i}^{2}e^{2}}\right)}^{\frac{1}{2}}$, where ɛ is the liquid permittivity, *k*_B_ is the Boltzmann constant, *T* is the liquid temperature, *e* is the unit charge, *c*_i0_ is the bulk concentration, and *z*_i_ is the valence of ionic species] (*36*, *41*, *42*) reduces from around 1 μm to 10 nm, and the Debye time [$\tau_{D}=\lambda_{D}^{2}/(D_{+}+D_{-})$, where *D_+_* and *D_−_* are the diffusion coefficients of the cations and anions, respectively] (*37*, *40*) decreases notably from around 60 μs to 30 ns. Please refer to table S1 for the parameters adapted in the calculation. In addition, at low concentrations, the growing conductivities of the solutions and shortened rising time of the corresponding peak voltages (tables S3 and S4) also contribute to the increased voltages. Further raising the ion concentration would reduce the surface charge density on surface of PTFE film (fig. S6), and thus, lead to a reduced *V*_peak_ (*12*, *43*). We note that the ion concentration corresponding to the maximum output voltage is notably higher than that revealed in other systems (*41*, *44*) and attribute the deviation to the different dielectric materials adapted and the way droplets and the solid surface interact. Considering the performance of tap water droplets are comparable to those of 1 mM NaCl solution in our experiments (tables S3 and S4), tap water is adapted in the following experiments due to its low cost and easy availability.

**Fig. 3.**
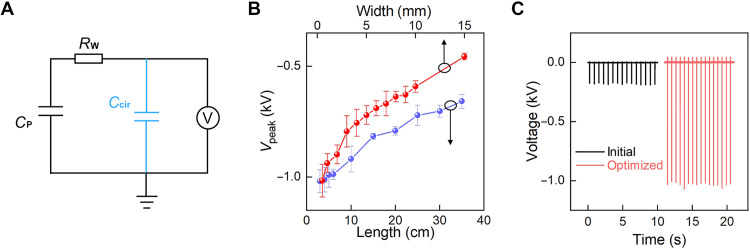
Device optimizations. (**A**) Equivalent electrical circuit of the system with consideration of a circuit capacitance *C*_cir_, *C*_1_, and *C*_2_ are ignored. (**B**) *V*_peak_ dependence on the top-electrode width (red color, the length of top electrode is fixed to 5 cm) and length (violet color, the width of top electrode is fixed to 0.5 mm). Measurements were conducted with a homemade voltage probe with minimized input capacitance. (**C**) The voltage increases from nearly 200 V to over 1000 V via circuit optimization. The size of the top electrode for the nonoptimized device is 10 cm (length) × 2 mm (width), and the circuit capacitance (*C*_cir_) is 29.18 pF, which is optimized to a top-electrode size of 5 cm × 0.5 mm and a *C*_cir_ of 1.05 pF.

Besides the microsecond contact dynamics, *C*_cir_ is also unavoidable but usually neglected, as highlighted by blue color in Fig. 3A. It consists of parasitic capacitance between the overlapped electrodes, stray capacitance between the top electrode and the Earth ground, and input capacitance of the measurement circuit. While considering the presence of *C*_cir_, *V*_peak_ becomes $V_{\mathrm{peak}}=\frac{C_{P}}{C_{P}+C_{\mathrm{cir}}}\cdot U_{0}=\frac{Q}{C_{P}+C_{\mathrm{cir}}}$, where *Q* is the charge stored in *C*_P_ initially. Note that *C*_2_ is ignored here for ease of analysis and because of the optimized contact dynamics with *C*_2_ notably larger than *C*_cir_ and *C*_P_. It is obvious that *V*_peak_ increases with the reduction of *C*_cir_ while keeping *C*_P_ constant, which indicates that the circuit capacitance also has a notable influence on *V*_peak_. Ideally, when *C*_cir_ is notably smaller than *C*_P_, the upper limit of the output voltage is around 1230 V for our devices, taking *Q* = *q*_0_ × *S* = 7.8 ± 0.67 nC and *C*_P_ = 6.49 pF (*S* ≈ 2.2 cm^2^).

The dependence of *V*_peak_ on the circuit capacitance *C*_cir_ guides the following device optimization to improve *V*_peak_. First, the parasitic capacitance between overlapped electrodes is reduced through adapting a narrow Au wire as the top electrode, which is placed close to the edge of the bottom electrode. As shown in Fig. 3B, while reducing its width from 15 to 0.5 mm with a corresponding parasitic capacitance of 13.28 and 0.44 pF, respectively, the output voltage is doubled in *V*_peak_. Similarly, *V*_peak_ increases with the decrease in top electrode length, as the electrode-ground stray capacitance is notably reduced. We also adapted a homemade oscilloscope probe in all these electrical measurements with a reduced input capacitance (see fig. S7). Through minimizing *C*_cir_, in combination with optimizing the microsecond contact dynamics, *V*_peak_ was improved to 1200 V, approaching the aforementioned upper limit, as shown in Fig. 3C.

Moreover, the output voltage could be further enhanced by increasing the surface potential of PTFE film, *U*_0_, according to Eq. 1. We have made efforts to raise *U*_0_ through increasing the surface charge density and realized an output sparking potential of more than 2000 V by surface charge injection, but it degraded notably during cyclic operation due to the poor stability of the injected surface charge (see fig. S9). Steady output of higher *V*_peak_ requires the development of robust charge injection approaches. It is worth mentioning that the devices without charge injection demonstrate a robust output performance for over 7000 cycles, as shown in fig. S10. In addition, the output current of our device is among the highest level, with the peak currents around 0.06, 1.5, and 2.3 mA for deionized water, 0.6 M NaCl, and tap water, respectively (fig. S11). The energy conversion efficiency is determined to be 2.7% (fig. S12).

The aforementioned strategy is also effective for devices of other configurations, such as device with a configuration shown in fig. S13, which has only one electrode on top of the PTFE film. The open-circuit *V*_peak_ of such a device can also be notably improved from 45 to 300 V through improving the formation rate of *C*_2_ and minimizing *C*_cir_.

**Fig. 4.**
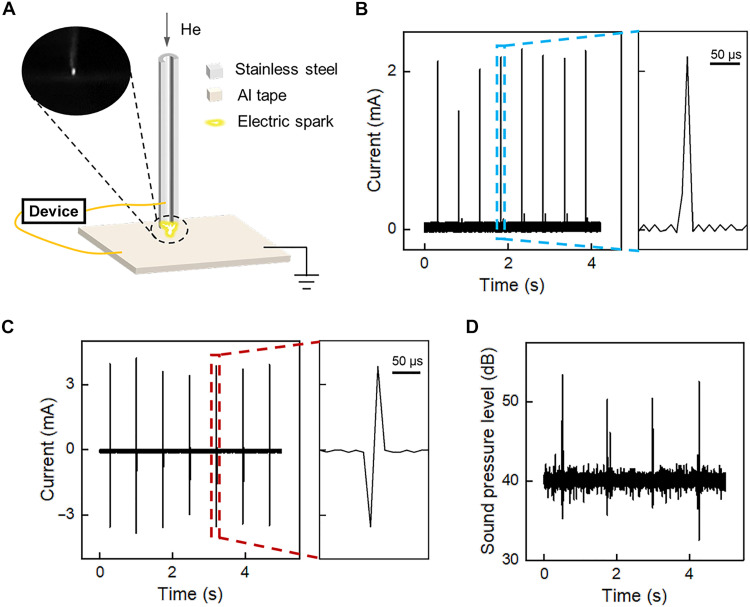
Gas ionization driven by sparking potential. (**A**) Schematic of the experimental setup for high-voltage discharge. Inset is a photograph of the microspark-induced light emission. (**B**) Typical current pattern of corona discharge at a large electrode-ground distance of 1 mm. The right panel is the enlarged view of the region marked by blue dashed rectangle. (**C**) Typical current pattern of microspark discharge while the tip electrode is placed close to the ground plate for a higher electric field. The right panel is the enlarged view of the region marked by the red dashed rectangle. The distance between tip electrode and ground plate is 0.3 mm. (**D**) Sound pressure level recorded while microspark discharge happened.

The ability to produce sparking potential of kilovolt level by a falling water droplet paves the way to high-voltage applications. Here, we successfully demonstrated that it is feasible to ionize helium gas at atmosphere pressure and produce microsparks by using an optimized device as the pulsed power source. As depicted in Fig. 4A, the high-voltage discharge setup consists of an Al plate as the ground electrode and a stainless-steel capillary tube with a diameter of 0.40 mm as the high-voltage tip electrode, through which helium gas flows at atmosphere pressure. The Al plate and capillary tube are connected to the bottom and top electrodes of the device, respectively.

The discharge behavior highly depends on the gap distance between these two electrodes. For a large gap distance of 1 mm, the main discharge behavior is corona discharge, during which the ionization of gas happens only in local space surrounding the tip electrode. It is because that the electric field in the gap is uneven and only electric field near the tip could exceed the dielectric strength of helium gas due to the tip-enhanced electric field (*45*, *46*). The typical current pattern was presented in Fig. 4B, in which the peak current is around 2 mA.

While reducing the gap distance, gas between the two electrodes can be fully ionized because of the increased electric field strength and microspark discharge happens along with bright light emission as that shown in inset of Fig. 4A (*47*, *48*). The typical current pattern for microspark discharge at a gap distance of 0.3 mm is shown in Fig. 4C. The peak current is 3.5 mA in average, notably higher than that of corona discharge due to the high conductivity of the ionized gas path. In contrast to the corona discharge, two current pulses of opposite polarity appear, corresponding to the rising and falling edges of the voltage pulse during microspark discharge (*49*).

Besides light emission, the microspark discharge is also accompanied by squeaks as the gas pressure changes during the instantaneous release of joule heat. The change of sound pressure level induced by microspark discharge could reach 13 dB, as shown in Fig. 4D, which can be easily heard. In a controlled experiment using a high-voltage source meter as the power supply, the threshold voltage for microspark discharge at the same gap distance is determined to be around 900 V, further verifying the ultrahigh amplitude of the sparking potential.

**Fig. 5.**
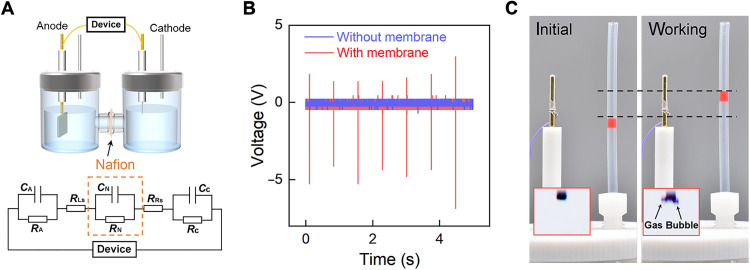
Water splitting directly powered by falling water droplets. (**A**) Schematic of the water electrolysis cell. In equivalent electrical circuit at the bottom panel, *C*_A_, *C*_C_, *R*_A_, and *R*_C_ are capacitances and impedances of anode and cathode, respectively. *C*_N_ and *R*_N_ are the capacitance and impedance of Nafion film, if there is. *R*_Ls_ and *R*_Rs_ are impedances of the left electrolyte and right electrolyte, respectively. (**B**) Output voltage for systems with and without the membrane. (**C**) Height lift of a capillary liquid of red color in a tube connected to the sealed cell due to the hydrogen generation, as well as photographs of electrode before and after water splitting, respectively.

The sparking potential is also high enough to drive the electrolysis of water directly in a cell with reduced capacitive load. As shown in Fig. 5A, the electrolysis cell consists of a ruthenium oxide–coated carbon paper and Pt wire serve as the anode and cathode, respectively, 1 M H_2_SO_4_ solution as the electrolyte. A Nafion film was used as the proton exchange membrane to separate the anode part and cathode part to reduce the load capacitance (fig. S14). The membrane can be regarded as a capacitance and a resistor in parallel, which are connected to the electrolyte in series, as illustrated in the bottom panel of Fig. 5A. Due to the EDL capacitance at the electrode/electrolyte interface being orders of magnitude higher than the device capacitance, the output voltage is limited and cannot be even distinguished from the measurement noise, due to the EDL capacitance at the electrode/electrolyte interface is orders of magnitude higher than the device capacitance. Through inserting the Nafion film, the output peak voltage reaches 5 V (Fig. 5B), which is large enough to directly drive the water splitting and generation of hydrogen and oxygen gases (see Fig. 5C).

## DISCUSSION

In summary, we presented an in-depth understanding of the liquid-solid interface dynamics at both the macroscale and microscale. The spreading of water droplet on the PTFE electret film firstly converts the mechanical energy to electrical energy stored at the interface, which can be simply regarded as a charging process of an interfacial capacitor. Following contacting and movement of the EDL boundaries across the top electrode edge cause the release of the stored energy instantaneously, i.e. discharging of the capacitor, giving rise to the sparking potential in the output circuit. A comprehensive model was then proposed to deeply explain and describe the working principle. It is shown that, a large droplet spreading area and an improved moving speed of the droplet boundaries, as well as a short relaxation time and thin thickness of EDL, are essential to achieve the ultrahigh sparking potential. Minimizing the circuit capacitance could further improve the peak amplitude. These optimizations lead to an ultrahigh sparking potential over 1200 V. This unprecedented kilovolt spark can enable gas ionization at atmospheric pressure and water splitting directly. It is also shown that increasing the surface potential of electret films can further notably improve the sparking potential.

## MATERIALS AND METHODS

### Device fabrication

A commercial PTFE film was cut into sheets of 5-cm–by–7-cm size and then washed by deionized water with following dry through nitrogen blow. The thickness of PTFE is 600 μm. An aluminum tape of desired width and length or an Au wire (10 μm in diameter for the optimized device) was set on the up side of the PTFE film to serve as the top electrode, and the back side of the PTFE film was fully covered by an aluminum tape to serve as the bottom electrode. An Al_2_O_3_ ceramic substrate (3 cm by 5.5 cm by 0.1 cm) was used as a solid support for the as-fabricated device. For water splitting, a ruthenium oxide–coated carbon paper electrode was prepared by a drop-casting method (*50*–*52*). The catalyst ink was prepared by sonication of a mixture of 20 mg of ruthenium oxide powder (Aladdin Chemical Co. Ltd.; 99.9%) and 20 mg of conductive carbon dispersed in 1 ml of ethanol and 100 μl of 5 wt % Nafion solution for 2 hours. The as-prepared catalyst ink (30 μl) was dropped onto a carbon paper electrode (1 cm by 1 cm) several times and left to dry.

### Measurements

In this work, tap water droplets, 60 μl in volume, were released from an infusion tube with its end fixed at a height of 25 cm above the device. The device was placed with an inclined angle of 45° unless otherwise specified. The falling frequency of the droplets is around 1.5 Hz. The output voltage of the device was recorded by an oscilloscope (Siglent, SDS2352X Plus) equipped with a homemade probe with minimized input capacitance or a commercial probe of high attenuation (10×, 10 megohm). The homemade probe was carefully calibrated by a standard square wave signal before all the measurements. The surface potential of PTFE film was determined by a DC-stable electrostatic voltmeter (Trek, 344). Droplets dynamics were recorded with a high-speed camera (AMETEK, Phantom VEO 1310L) at the rate of 5000 frames/s. The snapshots recorded by the high-speed camera and the voltage signals induced by falling droplet were synchronized through dual-channel measurement of the voltage signal from the device and the trigger signal for the high-speed camera. The current was collected using the same oscilloscope coupled with a low-noise current-to-voltage preamplifier (Stanford Research System, SR570). The sound pressure level was collected using a sound level meter (Hongsheng, HS5660B). The electrochemical impedance spectroscopy was carried out using an electrochemical workstation (AMETEK, PARSTAT 3000A-DX) with 10-mV perturbation. During all these experiments, the relative humidity of the laboratory was kept around 50%, and the environment temperature was held at 23.0° ± 2°C.

### Quantification of energy conversion efficiency

The energy conversion efficiency is calculated as η = *E*_out_/*E*_in_, and, here, $E_{\mathrm{out}}=\int_{t_{\mathrm{on}}}^{t_{\mathrm{off}}}I^{2}Rdt$, *E*_in_ = *mgh*, where the droplet mass, *m*, is 6 × 10^−5^ kg; the gravitational acceleration, *g*, is 9.8 m/s^2^; the releasing height of the droplet, *h*, is 0.25 m; *I* is the instantaneous current; and the resistance of the external load, *R*, is 30 megohm. For our device, the mechanical energy *E*_in_ carried by a tap water droplet was estimated to be 1.47 × 10^−4^ J, and the generated electrical energy *E*_out_ was calculated to be 4.03 × 10^−6^ J, and the corresponding energy conversion efficiency is approximately 2.7%.
