# Supplementary material for: Sparking potential over 1200 V by a falling water droplet
Source: Sci Adv. 2023 Nov 15;9(46):eadi2993. doi: 10.1126/sciadv.adi2993 (PMC10651119; doi:10.1126/sciadv.adi2993)
Supplement: Supplementary file 1 — Supplementary Text Figs. S1 to S15 Table S1 to S4 [file sciadv.adi2993_sm.pdf]

Science Advances
AAAS

# Supplementary Materials for

## Sparking potential over 1200 V by a falling water droplet

Luxian Li *et al.*

Corresponding author: Jun Yin, yinjun@nuaa.edu.cn; Wanlin Guo, wlguo@nuaa.edu.cn

*Sci. Adv.* **9**, eadi2993 (2023)
DOI: 10.1126/sciadv.adi2993

**This PDF file includes:**

Supplementary Text
Figs. S1 to S15
Table S1 to S4

**Supplementary Text**

The calculation of $C_{cir}$, $C_P$ and $C_2$

In detail, $C_P$ is the geometry capacitance between the water droplet and the bottom-electrode, thus $C_p = \varepsilon_p \cdot S/d$, where $S$ is the area of the spreading droplet while it just touches the top electrode and was determined from the snapshots captured by high-speed camera (as shown in Fig. 2D), $\varepsilon_P$ and $d$ are dielectric constant of PTFE ($2\varepsilon_0$) and thickness of the PTFE film (600 μm), respectively.

$C_2$ is the electrical double layer (EDL) capacitance between the water and top-electrode at the moment that the output voltage arrives peak value. It is expressed as $C_2 = t_{rising} \frac{dS_{overlap}}{dt} \cdot \frac{\varepsilon_2}{\lambda_D} \cdot (1 - e^{\frac{-t}{\tau_D}})$ (Equation (3) in the manuscript), where $S_{overlap}$ is the overlap area between the droplet and the top-electrode and was determined from the snapshots captured by high-speed camera, $t_{rising}$ is the rising time of the pulsed voltage signal and was read from the electrical signal, $\tau_D$, $\lambda_D$ and $\varepsilon_2$ are Debye time of $C_2$ (a character to describe the relaxation time of EDL, 30 ns), Debye length of $C_2$ (a character to describe the thickness of EDL, 10 nm), dielectric constant of water (78 $\varepsilon_0$), respectively.

$C_{cir}$ is obtained through a series of tests of the output voltage while the device is connected to a variable capacitor ($C_{out}$) in parallel. The equivalent circuit is shown in Fig. S8A, and $C_{cir}$ was determined through following fitting to $V_{peak} = \frac{Q}{C_P + C_{cir} + C_{out}}$, which gives 29.18 pF here.

The determination of the circuit capacitance before and after optimization

To verify that the improved $V_{peak}$ is attributed to the reduction of $C_{cir}$ and evaluate the $C_{cir}$ after optimization, we investigated the dependence of $V_{peak}$ on the external capacitor connected in parallel with the device, $C_{out}$. The equivalent circuit is shown in Fig. S8A. It is shown in Fig. S8B that $V_{peak}$ decays rapidly from over 1,000 V to close to zero as $C_{out}$ increases from 0 to 1 μF. A circuit capacitance of 1.05 pF is determined by fitting the experimental data with the function, $V_{peak} = \frac{Q}{C_P + C_{cir} + C_{out}}$. Similarly, a notably larger circuit capacitance of 29.18 pF was determined for the circuit before optimization, see Fig. S8C.

The derivation of the Equation (1)

As shown in Fig. S15 ($C_1$ has been ignored here based on the discussion in the main text), since $C_2$ and $C_{cir}$ are in series connection, for convenience, we let $C_{equ}$ instead of $C_2$ and $C_{cir}$, and $C_{equ} = \frac{C_2 \cdot C_{cir}}{C_2 + C_{cir}}$. Once the droplet touches the top electrode, owing to the presence of a potential difference between $C_P$ and $C_{equ}$, charges, $Q_T$, has to transfer from $C_P$ to $C_{equ}$ until the circuit reaches equilibrium, i.e., $U_{equ} = U_P$, where $U_{equ}$, $U_P$ are the voltages across $C_{equ}$ and $C_P$, respectively.

And then the transferred charge,

$$Q_T = C_{equ} \cdot U_{equ} = C_P \left(U_0 - U_P\right) = C_P \left(U_0 - U_{equ}\right),$$

therefore,

$$U_{equ} = \frac{C_P}{C_{equ} + C_P} \cdot U_0,$$

$$Q_T = C_{equ} \cdot U_{equ} = \frac{C_{equ} \cdot C_P}{C_{equ} + C_P} \cdot U_0 \,,$$

where $U_0$ is the initial voltage across $C_P$.
Thus, the peak output voltage

$$V_{peak} = \frac{Q_T}{C_{cir}} = \frac{C_{equ} \cdot C_P}{\left(C_{equ} + C_P\right) \cdot C_{cir}} \cdot U_0 = \frac{1}{1 + C_{cir} / C_P + C_{cir} / C_2} \cdot U_0 \,.$$

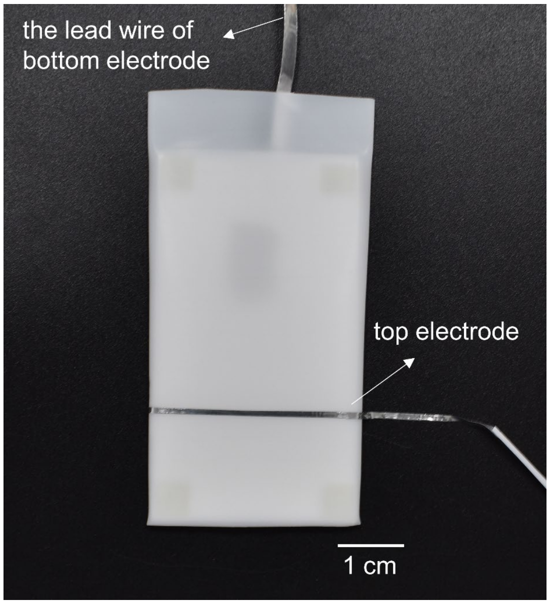


**Fig. S1. Photograph of the device.**

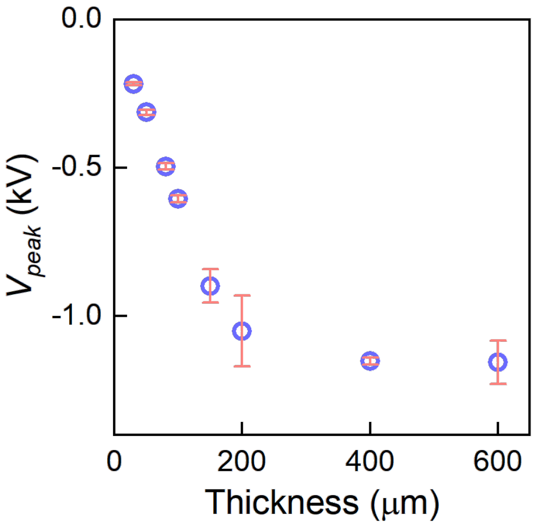


**Fig. S2. Dependence of $V_{peak}$ on the thickness of PTFE film.**

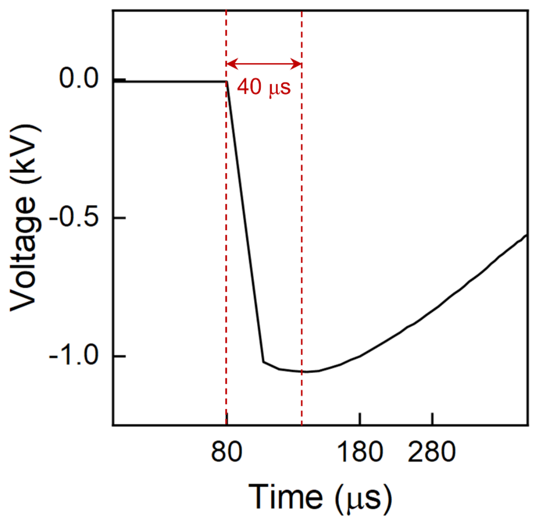


**Fig. S3. The enlarged view of the sparking potential focusing on microsecond rising time.**

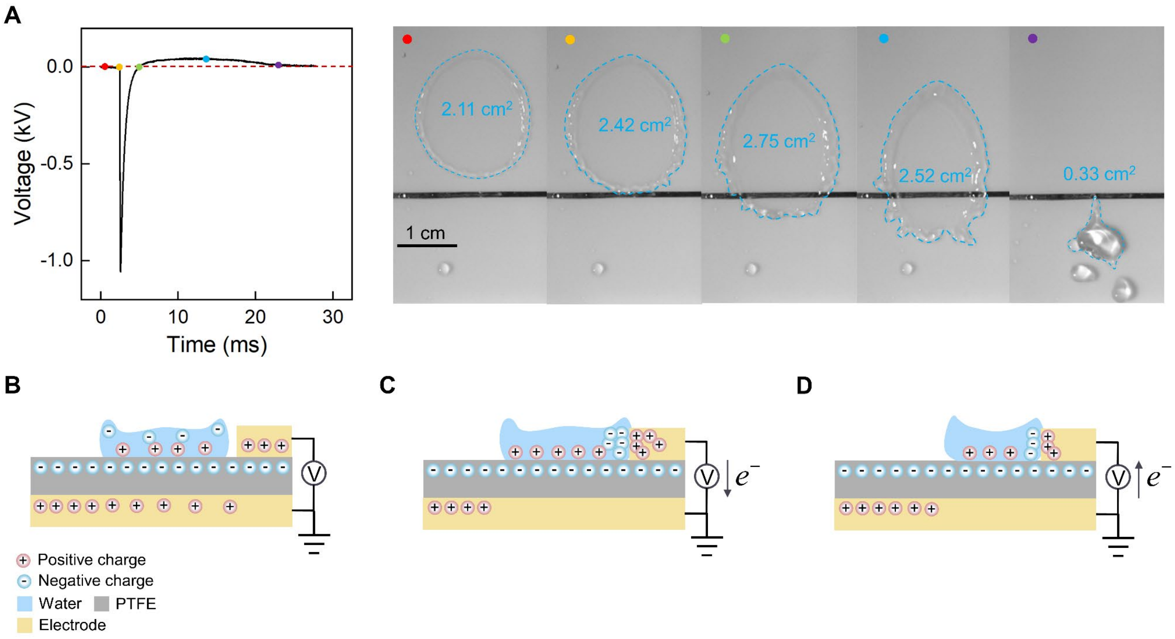


**Fig. S4. Origination of the positive peak voltage.** (**A**) The signal of one single peak voltage and the corresponding snapshots of different stages. Blue dashed line indicates the boundary of the droplet. (**B**)-(**D**) Schematic of charge distribution in the device before (**B**) and after (**C**) the spreading droplet touches the top-electrode, and when the droplet area shrinks (**D**).

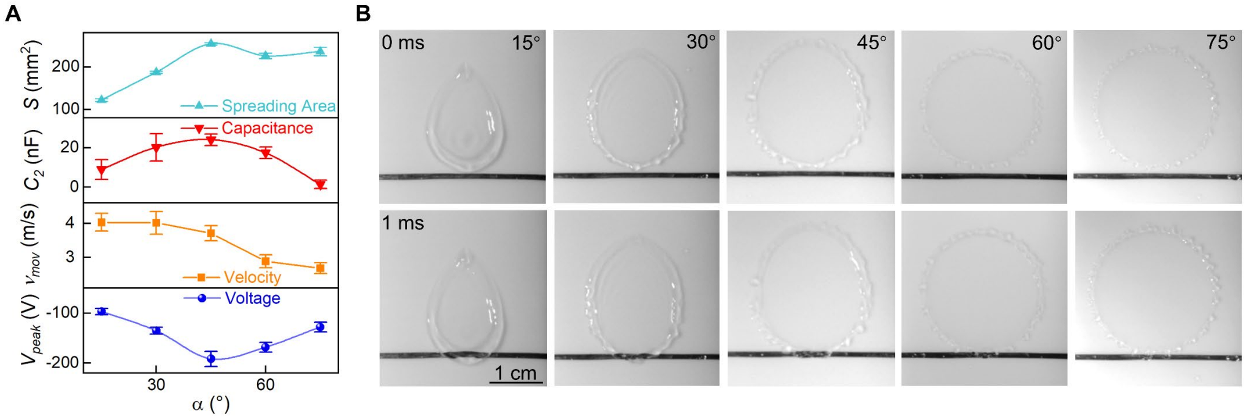


**Fig. S5. Dependence of $V_{peak}$ on the inclined angle, α, and the mechanism.** (**A**) Droplet spreading area, *S*, the capacitance of $C_2$, the velocity of moving boundary, $v_{mov}$, peak output voltage, $V_{peak}$, dependence on $\alpha$. These values were extracted from snapshots just after the droplet touches the top electrode. (**B**) Typical snapshots for a droplet just before and after the droplet touches the top electrode of devices placed at 15°, 30°, 45°, 60°, 75°.

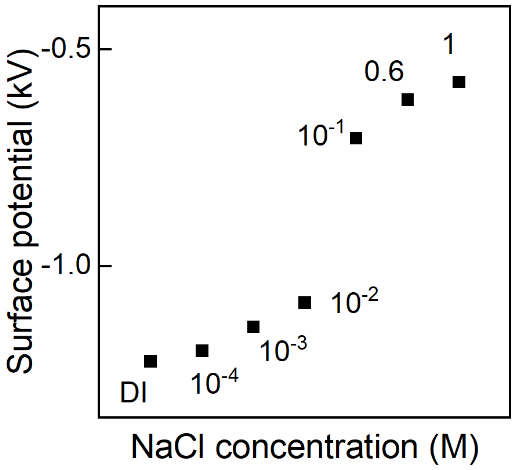


**Fig. S6. Dependence of surface potential on the NaCl concentration.**

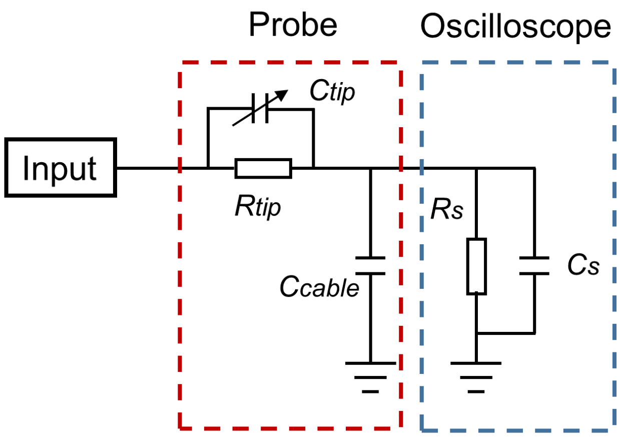


**Fig. S7. The measurement circuit with a homemade voltage probe,** in which, $R_{tip}$ = 99 Mohm, $R_s$ = 1 Mohm, $C_{tip} < 1$ pF, $C_{cable} \approx 0$ pF, and $C_s$ = 17 pF, respectively.

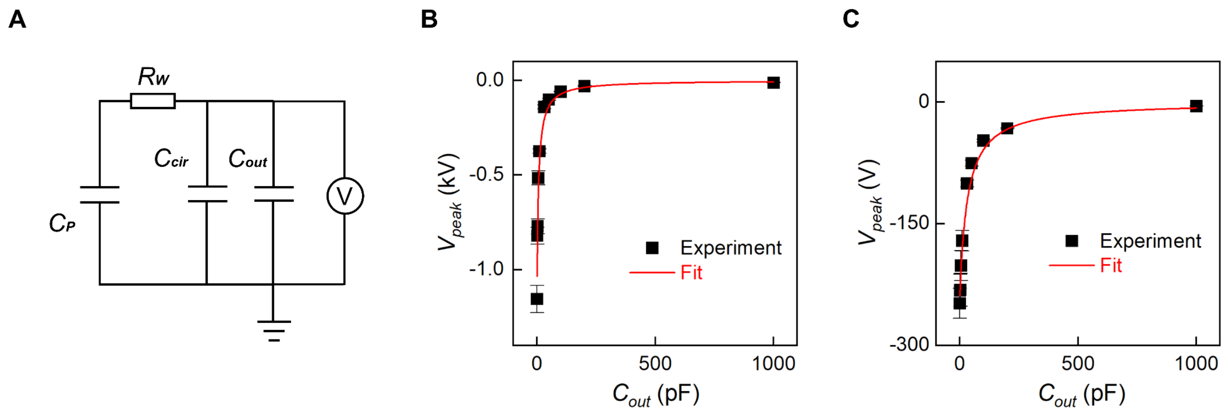


**Fig. S8. Dependence of $V_{peak}$ on the external capacitor connected in parallel with the device, $C_{out}$.** (**A**) Equivalent electrical circuit of the system with an external capacitive load, $C_{out}$. (**B**) Peak voltage dependence on $C_{out}$ for the system with minimized circuit capacitance. (**C**) Peak voltage dependence on $C_{out}$ for device before the circuit optimization.

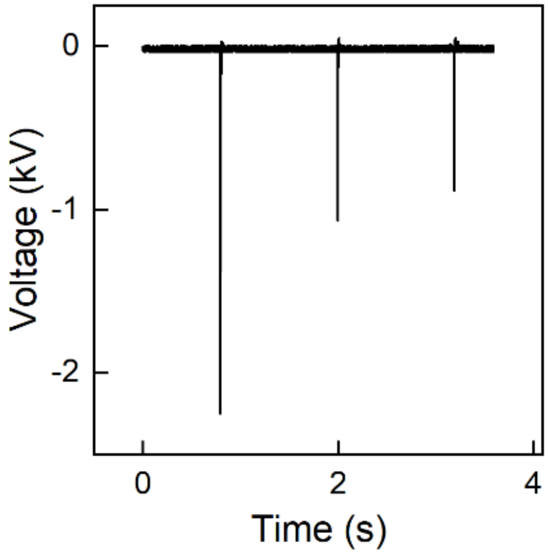


**Fig. S9. The output voltage of a transistor-inspired device after surface charge injection via an air ionization gun.**

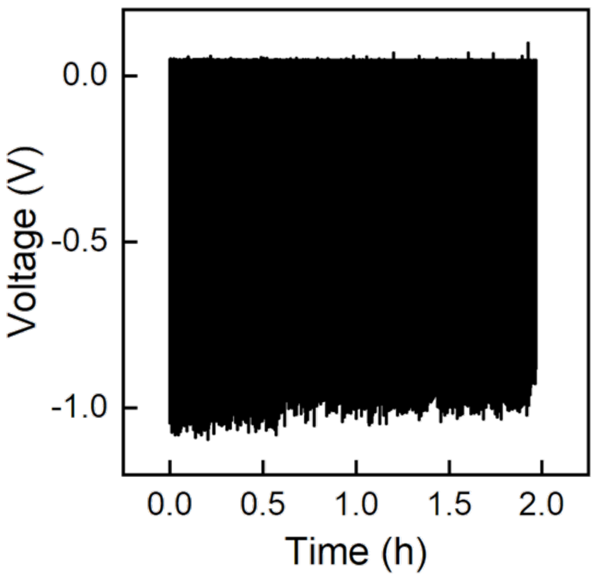


**Fig. S10. Long-term stability of the device.**

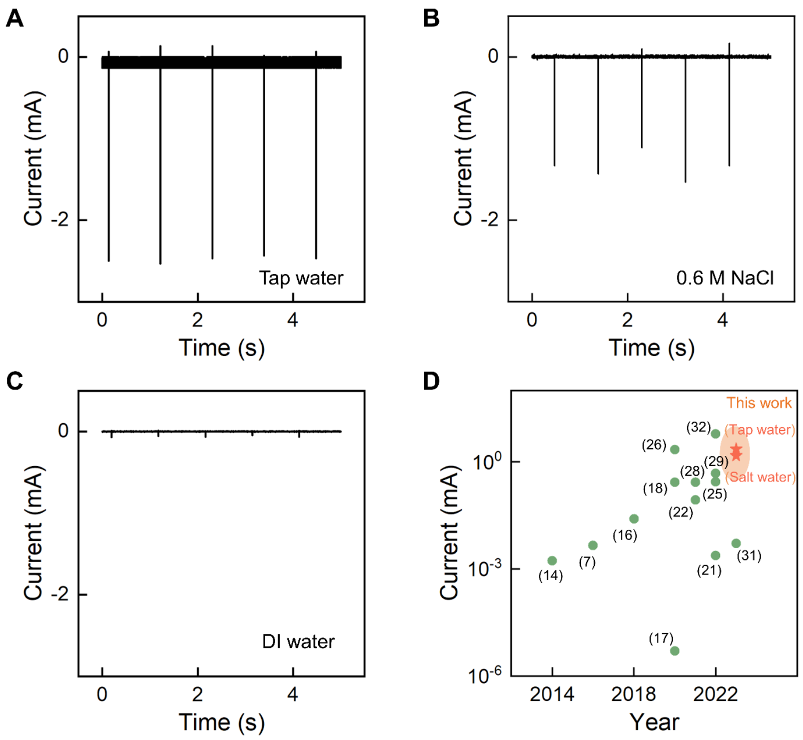


**Fig. S11. The current output of the device.** The short-circuit current of the device for tap water (**A**), 0.6 M NaCl (**B**) and deionized (DI) water (**C**). (**D**) Output peak current driven by falling water droplets reported previously and in this work.

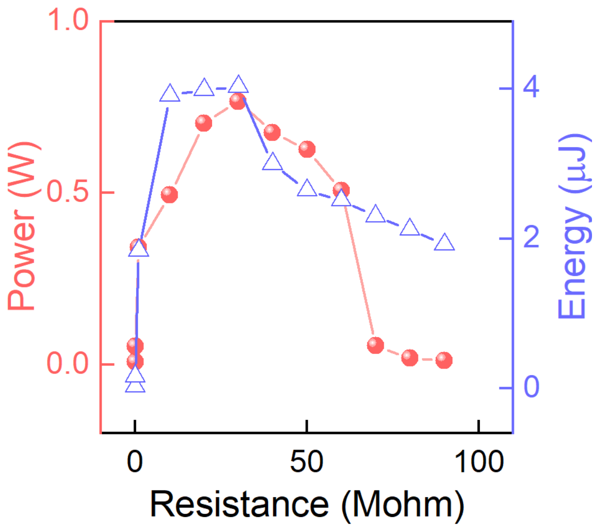


**Fig. S12. Dependence of the output power and energy on the load resistance.**

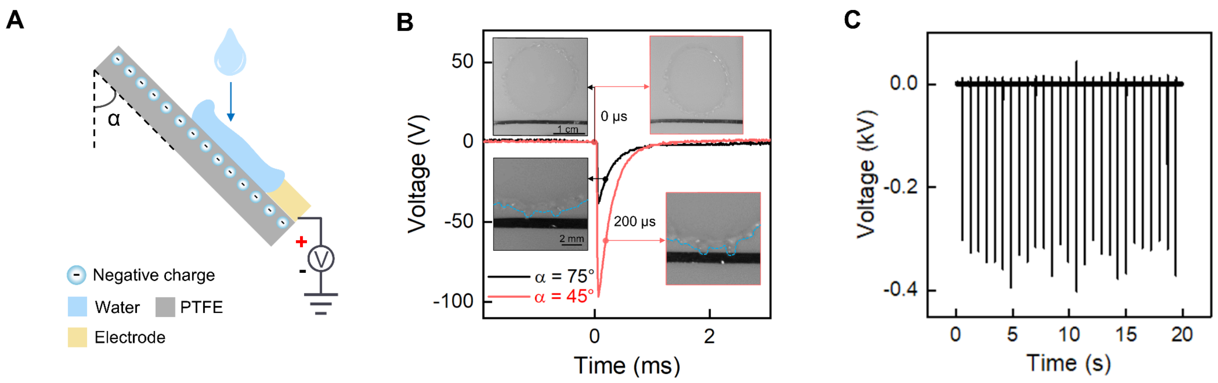


**Fig. S13. Configuration and output performance of a single-top-electrode device.** (**A**) Schematic of the single-top-electrode device configuration, not to scale. (**B**) Typical output voltage signals for the device placed at α = 75°, 45° and corresponding snapshots just before and after the spreading droplet touches the electrode. The blue dashed line indicates the droplet boundary. (**C**) Output voltage of a single-top-electrode device with minimized circuit capacitance.

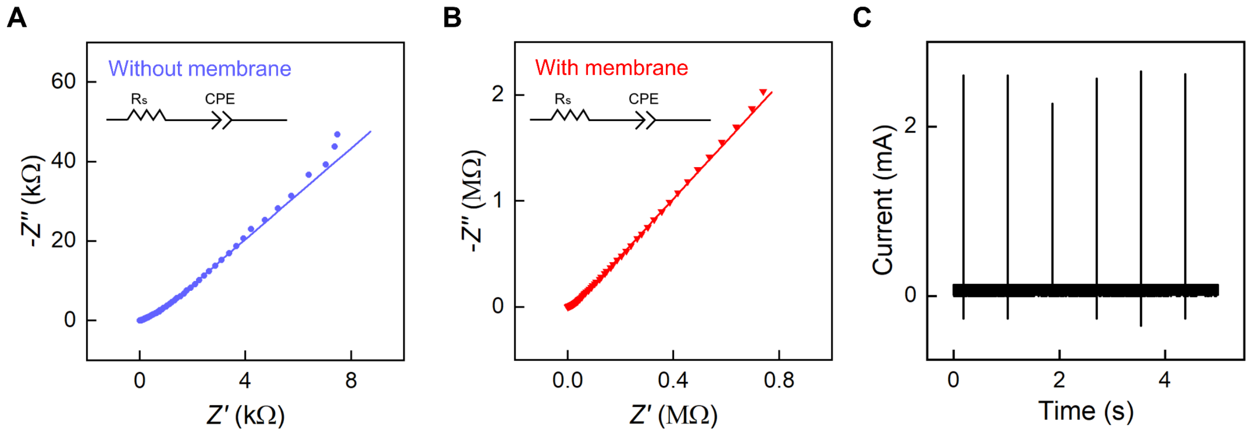


**Fig. S14. The electrochemical impedance spectroscopy (EIS) Nyquist curves without (A) and with (B) the Nafion membrane at 0 V, respectively.** The fitted values are $R_s$ = 425.5 ohm, *CPE* = 4.0325 μF in (A); $R_s$ = 21476 ohm, *CPE* = 0.11147 μF in (B). (**C**) Working current of the system.

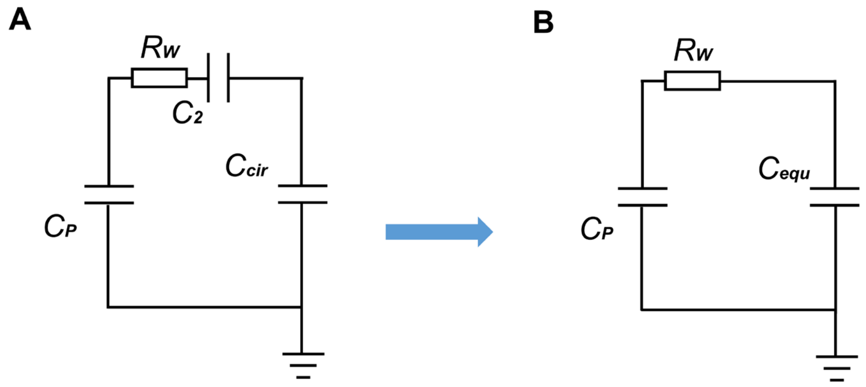


**Fig. S15. The full equivalent circuit (A) and simplified equivalent circuit (B).**

**Table S1. The parameters used for calculating Debye length and Debye time.**

| Parameter | | Value | Unit | Note |
|---|---|---|---|---|
| $\varepsilon$ | | 8.85×10$^{-12}$×78 | F/m | |
| $k_B$ | | 1.38×10$^{-23}$ | J/K | |
| $T$ | | 273.15+25 | K | |
| $e$ | | 1.6×10$^{-19}$ | C | |
| $c_0(H^+)$ | (DI Water) | 1×10$^{-4}$ mol | $m^{-3}$ | |
| $z(H^+)$ | | 1 | | |
| $c_0(OH^-)$ | (DI Water) | 1×10$^{-4}$ mol | $m^{-3}$ | |
| $z(OH^-)$ | | 1 | | |
| $c_0(Na^+)$ | (1 mM NaCl) | 1 mol | $m^{-3}$ | |
| $z(Na^+)$ | | 1 | | |
| $c_0(Cl^-)$ | (1 mM NaCl) | 1 mol | $m^{-3}$ | |
| $z(Cl^-)$ | | 1 | | |
| $D(H^+)$ | | 9.31×10$^{-9}$ | $m^2s^{-1}$ | Refer to the |
| $D(OH^-)$ | | 5.27×10$^{-9}$ | $m^2s^{-1}$ | thermodynamic |
| $D(Na^+)$ | | 1.33×10$^{-9}$ | $m^2s^{-1}$ | database |
| $D(Cl^-)$ | | 2.03×10$^{-9}$ | $m^2s^{-1}$ | "phreeqc.dat" |
| $N_A$ | | 6.02×10$^{23}$ | | |

* Parameters with yellow background are used for DI water, and those with blue background are used for 1 mM NaCl.

**Table S2. The parameters used for calculating $C_2$.**

| Parameters | Value | Unit | Note |
|---|---|---|---|
| $t_{rising}$ | 40 | μs | Refer to Fig. S3 |
| $dS_{overlap}/dt$ | 0.43374/120 | $mm^2/\mu s$ | Refer to Fig. 2D |
| $\varepsilon_2$ | $78\times 8.85\times10^{-12}$ | F/m | |
| $\lambda_D$ | 10 | nm | Values for 1 mM NaCl were taken due to the similar conductivity |
| $\tau_D$ | 30 | ns | |

**Table S3. Physical and chemical properties of NaCl solution and tap water.**

| NaCl Concentration (mol/L) | 0 (DI) | $10^{-6}$ | $10^{-5}$ | $10^{-4}$ | $10^{-3}$ | $10^{-2}$ | $10^{-1}$ | 0.6 | 1 | Tap |
|---|---|---|---|---|---|---|---|---|---|---|
| conductivity (μS/cm) | 0.78 | 0.92 | 1.79 | 11.5 | 156 | 1084 | 10630 | 42200 | 75700 | 342 |
| pH | 8.56 | 8.55 | 8.74 | 8.67 | 7.86 | 7.05 | 6.25 | 5.43 | 5.21 | 7.92 |

**Table S4. The rising time of the peak voltage with NaCl solution and tap water.**

| NaCl Concentration (mol/L) | 0 (DI) | $10^{-6}$ | $10^{-5}$ | $10^{-4}$ | $10^{-3}$ | $10^{-2}$ | $10^{-1}$ | 0.6 | 1 | Tap |
|---|---|---|---|---|---|---|---|---|---|---|
| rising time (μs) | 740 | 380 | 350 | 150 | 40 | 17 | 7 | 5 | 5 | 40 |
